# Blurred image restoration using knife-edge function and optimal window Wiener filtering

**DOI:** 10.1371/journal.pone.0191833

**Published:** 2018-01-29

**Authors:** Min Wang, Shudao Zhou, Wei Yan

**Affiliations:** 1 College of Meteorology and Oceanography, National University of Defense Technology, Jiangsu Province, PR of China; 2 Collaborative Innovation Center on Forecast and Evaluation of Meteorological Disasters, Nanjing University of Information Science and Technology, Jiangsu Province, PR of China; Mar Ephraem College of Engineering & Technology, INDIA

## Abstract

Motion blur in images is usually modeled as the convolution of a point spread function (PSF) and the original image represented as pixel intensities. The knife-edge function can be used to model various types of motion-blurs, and hence it allows for the construction of a PSF and accurate estimation of the degradation function without knowledge of the specific degradation model. This paper addresses the problem of image restoration using a knife-edge function and optimal window Wiener filtering. In the proposed method, we first calculate the motion-blur parameters and construct the optimal window. Then, we use the detected knife-edge function to obtain the system degradation function. Finally, we perform Wiener filtering to obtain the restored image. Experiments show that the restored image has improved resolution and contrast parameters with clear details and no discernible ringing effects.

## Introduction

Camera movements might result in motion blur in captured images. The blurred image is usually modeled as a convolution between the original image and a known point spread function (PSF). Image restoration techniques are used to remove or minimize known degradations in an image. There are several classical image restoration methods, such as the iterative Lucy–Richardson algorithm and the non-iterative Wiener algorithms [1–2]. Several complex methods such as the Bussgang algorithm [3] have also been proposed. In [4], an adaptive restoration method to adaptively correct retinal images is proposed. This is performed by using deconvolution to remove the residual wave-front aberrations and provide an improvement over the Wiener filter with respect to the quality of restoration. An efficient technique based on physical optics is presented in [5]. In this, space-variant blurs are restored by sectioning using modified Wiener filtering. In [6], an image reconstruction and restoration method using the simplified topological ε-algorithm is proposed. In [7], the generalized Hermitian and skew-Hermitian splitting (GHSS) iterative method is applied to the problem of image restoration.

A novel method is presented in [8] to estimate the depth map in an underwater scenario. The authors also describe a method to handle the presence of artificial illumination in underwater images. In [9], a novel Bayesian image restoration method that integrates Total Variation (TV) and Poisson singular integral (PSI) models is proposed. An image restoration method that combines the conditional gradient with the Tikhonov regularization technique is described in [10]. In [11], a new filtering process based on adaptive diffusion function in the image restoration is discussed. A fast and automatic method for finding the direction and size of the blur is presented in [12]. In [13], a new variational model for joint multiplicative denoising and deblurring is proposed, which combines a total generalized variation filter and shearlet transform. A two-level domain decomposition method for image restoration is proposed in [14], which consists of an overlapping domain decomposition technique and a coarse mesh correction. A method for image restoration is presented in [15], which can adaptively determine the optimal norms for both fidelity term and regularization term in the (SR) restoration model. In [16], a fast half-quadratic method for image restoration and reconstruction is proposed. A class of upper and lower triangular splitting iteration algorithm for image restoration is proposed in [17]. In [18], an image restoration method for fiber-coupled image using space-variant impulse response characterization is proposed. A nonconvex and nonsmooth total generalized variation (TGV) model for image restoration is presented in [19], which can provide an even sparser representation of the variation of the image function than the traditional TGV model that uses convex L_1_ norm to measure the variation. A new two-step iterative shrinkage/ thresholding method for image restoration is proposed in [20], which exhibiting much faster convergence rate than IST for ill-conditioned problems. In [21], an image restoration method based on multi-scale patch, which imposes the very same prior on different scale patches extracted from the target image. A variational method for joint optical flow estimation and edge-aware image restoration is presented in [22]. A new approach of multi-frame super-resolution using diffusion registration and a nonlocal variational image restoration is proposed in [23]. In [24], a new image restoration method based on new adaptive anisotropic diffusion function is proposed, which is able to overcome the drawbacks of the traditional process. A novel optimization method for image restoration based on quadratic data fitting and smooth non-quadratic regularization is presented in [25]. A novel Laplacian-based visibility restoration method for real-world hazy scenes to effectively solve inadequate haze thickness estimation and alleviate color cast problems is proposed in [26]. Two image restoration methods using simultaneous sparse coding (SSC) and nonlocally centralized sparse representation are proposed in [27] and [28], respectively. In [29], an edge-preserving image restoration is proposed, which combines a masking operator that prevents wraparound artifacts.

In this paper, we present an image restoration technique based on the knife-edge function and optimal window Wiener filtering. First, we use the Prewitt edge detection operator and autocorrelation function to calculate the direction and scale of the motion-blur. Following this, we add the optimal window to the edge extension image. Then, we use the detected knife-edge function to obtain the system degradation function. Finally, we use Wiener filtering to obtain the restored image and truncate the border.

Experimental evaluations show that this method is effective in restoring images. The resolution and contrast parameters are improved, details are clear, and no ringing effects are observed. The proposed method exhibits superior performance in comparison with other techniques on the basis of metrics like the gray mean grads (GMG), peak signal-noise ratio (PSNR), and improvement signal-to-noise rate (ISNR).

The rest of the paper is organized as follows. Section 2 describes the principles of motion-blurred image degradation, restoration, and optimal window construction. Section 3 explains the restoration method proposed in this paper. Section 4 describes the experimental evaluation results. Section 5 concludes this paper.

## Theoretical foundations

### The principles of image restoration

**Fig 1:**
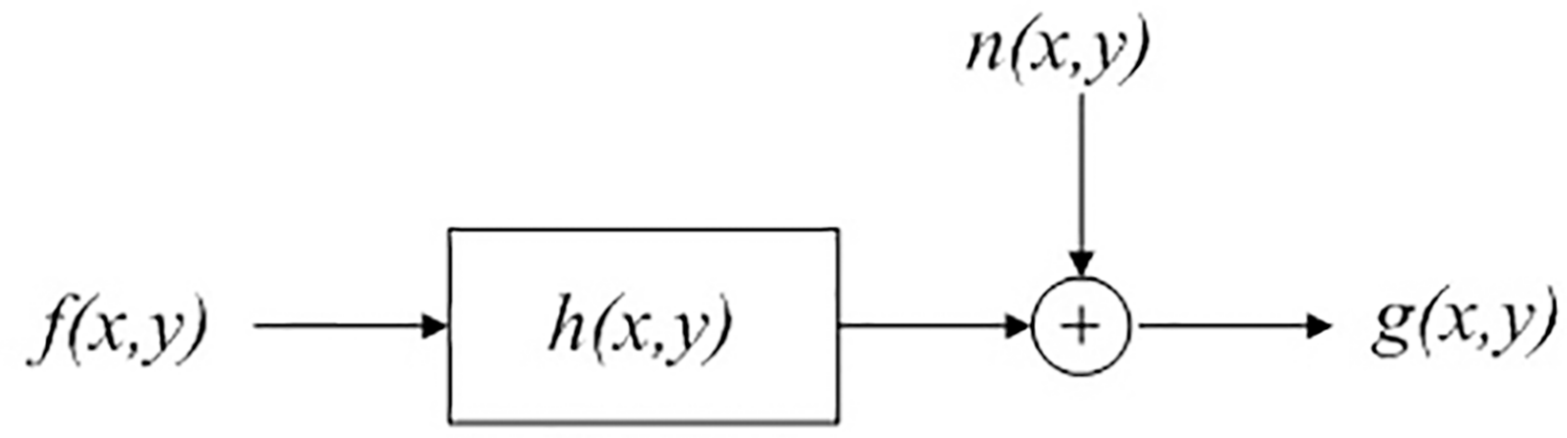
Mathematical model of image degradation.

The digital representation of a photographic image consists of a matrix that has information about the intensity levels of the individual pixels. In the linear space translation, a blurred image can be described using a two-dimensional convolution model, as shown in Fig 1.

g(x,y)=f(x,y)*h(x,y)+n(x,y) (1)

Here, *g*(*x*,*y*) represents the blurred image, *f*(*x*,*y*) refers to the original image, *h*(*x*,*y*) is the PSF or degradation function, and *n*(*x*,*y*) is random noise caused by the camera sensor. The notation “*” represents the convolution operation. On applying Fourier transformation to the above equation, we obtain the blurred model expression in the frequency domain based on the convolution theorem.

G(u,v)=F(u,v)⋅H(u,v)+N(u,v) (2)

where *G*(*u*,*v*), *F*(*u*,*v*), *H*(*u*,*v*), and *N*(*u*,*v*) are the two dimensional Fourier transformations of *g*(*x*,*y*), *f*(*x*,*y*), *h*(*x*,*y*), and *n*(*x*,*y*), respectively, and the operator “·” represents element-wise multiplication.

The PSF is determined by the length (*L*) and angle (*θ*) of the camera motion. Hence, we can construct a PSF or filtering function *H*(*u*,*v*) to restore the original image *f*(*x*,*y*).

$$f(x,y)=F^{-1}[\frac{G(u,v)-N(u,v)}{H(u,v)}]$$ (3)

The Wiener filter uses the follow filtering function.

$$\hat{F}(u,v)=\frac{1}{H(u,v)}\frac{\left|H(u,v)\right|}{|H(u,v){|}^{2}+k}G(u,v)$$ (4)

where |*H*(*u*,*v*)|^2^ = H*(*u*,*v*)*H*(*u*,*v*). H*(*u*,*v*) is the conjugate complex of *H*(*u*,*v*), and *k* is the constant signal-to-noise ratio chosen from 0.0001 to 0.01.

Wiener filtering takes into consideration the degradation function and noise statistical features, but it can produce the edge error.

### The principles of optimal window Wiener filtering

**Fig 2:**
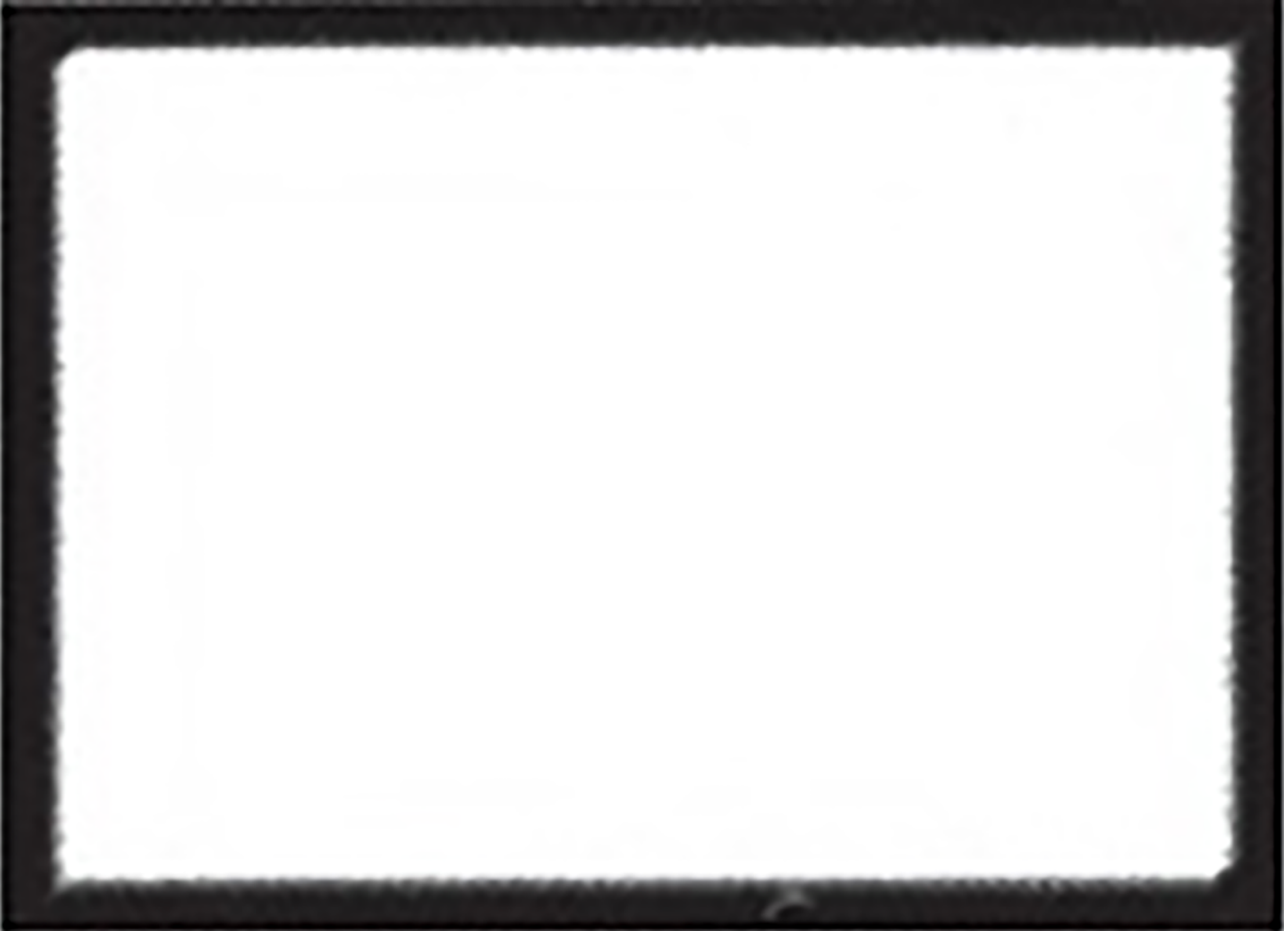
Optimal window.

**Fig 3:**
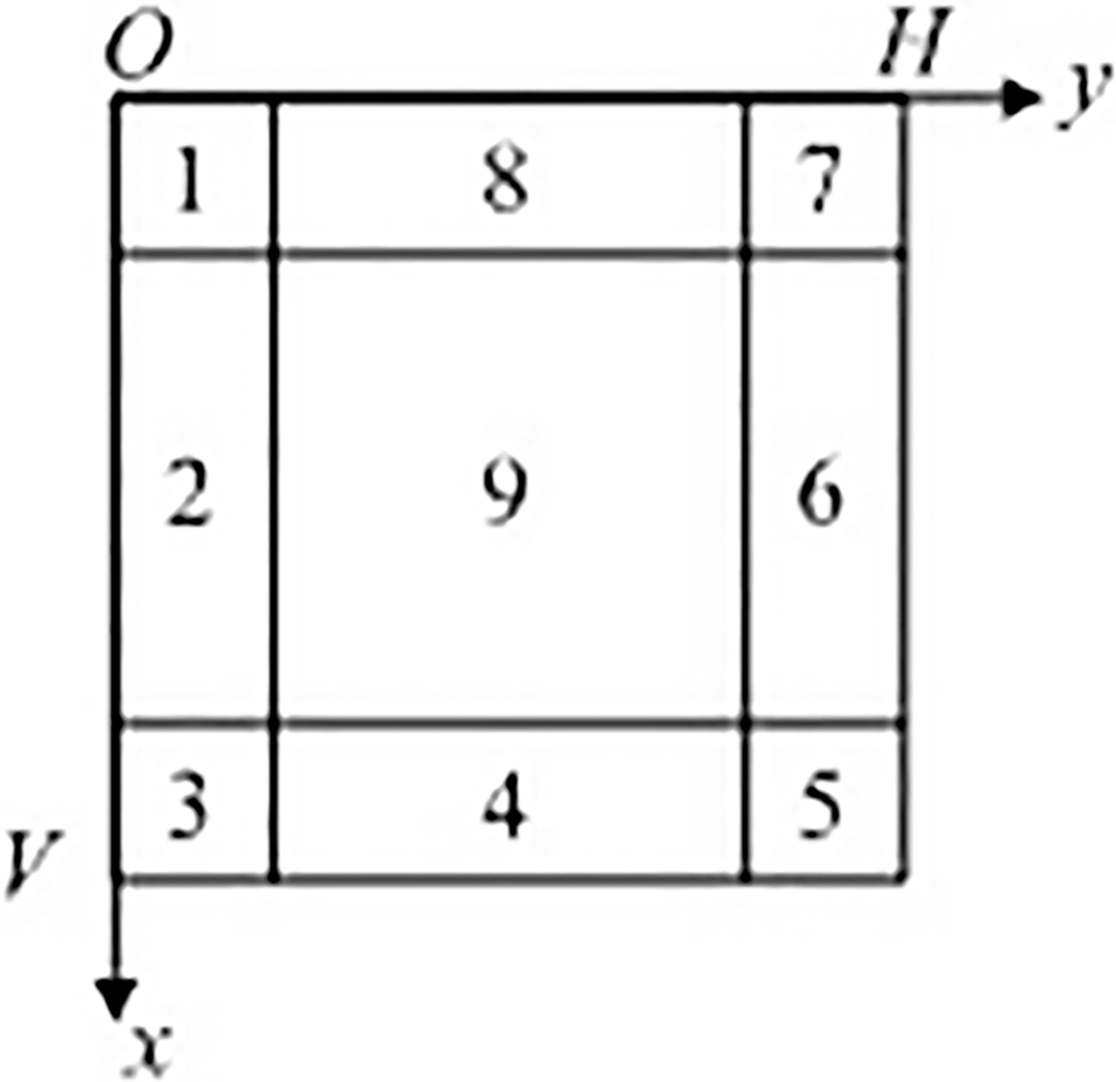
Optimal window region.

Optimal window Wiener filtering is used to suppress the edge error [30], as shown in Fig 2 and Fig 3. We calculate the window function *ω*(*x*,*y*) of each image region based on the amount of motion experienced by that segment. For optimal window regional range and coordinates parameter values, see Table 1. The window function *ω*(*x*,*y*) is used as the weighting factor while performing a Fourier transform of the motion-blurred image *g*(*x*,*y*).

$$\hat{f}(x,y)=g(x,y)*\omega (x,y)$$ (5)

$$\omega (x,y)=\left[\begin{array}{ccc} \sum_{m=0}^{x}\sum_{n=0}^{y}h(m,n) & \sum_{m=0}^{x}\sum_{n=0}^{P_{\mathrm{SFH}}^{}‑1}h(m,n) & \sum_{m=0}^{x}\sum_{{n=y+P}_{\mathrm{SFH}}^{}‑H}^{P_{\mathrm{SFH}}^{}‑1}h(m,n)\\ \sum_{m=0}^{P_{\mathrm{SFV}}^{}‑1}\sum_{n=0}^{y}h(m,n) & 1 & \sum_{m=0}^{P_{\mathrm{SFV}}^{}‑1}\sum_{{n=y+P}_{\mathrm{SFH}}^{}‑H}^{P_{\mathrm{SFH}}^{}‑1}h(m,n)\\ \sum_{{m=x+P}_{\mathrm{SFV}}^{}‑V}^{P_{\mathrm{SFV}}^{}‑1}\sum_{n=0}^{y}h(m,n) & \sum_{{m=x+P}_{\mathrm{SFV}}^{}‑V}^{P_{\mathrm{SFV}}^{}‑1}\sum_{n=0}^{P_{\mathrm{SFH}}^{}‑1}h(m,n) & \sum_{{m=x+P}_{\mathrm{SFV}}^{}‑V}^{P_{\mathrm{SFV}}^{}‑1}\sum_{{n=y+P}_{\mathrm{SFH}}^{}‑H}^{P_{\mathrm{SFH}}^{}‑1}h(m,n) \end{array}\right]$$ (6)

**Table 1:** Optimal window regional range and coordinates parameter values.

Region label	horizontal coordinate (*y-axis*)	Region label	vertical coordinate (*x-axis*)
1, 2, 3	[0,*P*_*SFH*_−2]	1, 8, 7	[0,*P*_*SFV*_−2]
4, 8	[*P*_*SFH*_−1,*H*−*P*_*SFH*_]	2, 6	[*P*_*SFV*_−1,*V*−*P*_*SFV*_]
5, 6, 7	[*H*−*P*_*SFH*_+1,*H*−1]	3, 4, 5	[*V*−*P*_*SFV*_+1,*V*−1]

The steps followed in the motion-blurred image restoration procedure using optimal window Wiener filtering are given below.

1. Compute the Fourier transformation $\bar{G}(u,v)$ of the windowed motion-blurred image *g*(*x*,*y*)*ω(*x*,*y*).
2. Assign PSF *h*(*m*,*n*) and compute its Fourier transformation *H*(*u*,*v*).
3. Compute the inverse Fourier transformation of $\frac{\bar{G}(u,v)H^{*}(u,v)}{{\left|H(u,v)\right|}^{2}+k}$ to obtain the restored image.

Wiener filtering using an optimal window can prevent edge errors effectively, but this technique also has its drawbacks. There is a L-shaped strip, at the right bottom corner, of width *P*_*SFH*_ and height *P*_*SFV*_, that exists due to the incompleteness of the image data. In this paper, we consider a two-dimensional extension to the edge of the image, such that the extension of width and height is consistent with the motion component *P*_*SFH*_ *and P*_*SFV*_. After performing the optimal window Wiener filtering, we truncate the edge extensions.

### Determination of PSF

According to the degradation model, the key to image restoration is understanding and estimating the PSF or *H*(*u*,*v*) of the imaging system accurately. From Eq (4), if *H*(*u*,*v*) is known, we can calculate $\hat{F}(u,v)$, and consequently *f*(*x*,*y*). In general, the PSF or *H*(*u*,*v*) is determined using prior knowledge or experimental information.

In real-life applications, the PSF is determined according to the image and motion features. It is difficult to obtain the clear point and line objects for a motion-blurred image. However, it is easy to obtain the straight edge image, and consequently the knife-edge function, of the object through edge detection. The knife-edge function has many advantages. First, there is no need for prior knowledge of the specific degradation model of the motion-blurred image, as we can use the knife-edge function to construct PSF and estimate the degradation function. Second, the knife-edge function is not sensitive to any specific form of motion-blurring, and hence is suitable for modeling various types of motion-blur.

A brief summary of the steps followed in the PSF computation process is given below.

1. Compute the direction of blurring, theta, by using the Prewitt operator to detect the motion trajectory of the edge feature.
2. Rotate the image such that the motion-blurred direction is parallel to the horizontal direction (*x*-axis).
3. Locate the maximum point of $\frac{\partial g}{\partial x}$ in the horizontal direction and assign this as the center pixel of the knife-edge function.
4. Use the center pixel point to construct the knife-edge function.
5. Compute the differential of the knife-edge function to get the line spread function (LSF) of the degradation system such that the PSF is the LSF along the *x*-axis direction.
6. Fourier transform the PSF to obtain the system degradation function *H*(*u*,*v*).

In order to improve the detection accuracy of the knife-edge function, we can detect the knife-edge function along several edges and compute the average gray value of the corresponding position.

## Proposed image restoration technique

The algorithm of the proposal presented in this paper is as follows (S1 Fig):

1. Use the method in [31] to eliminate noise from the image.
2. Compute the blurred direction *θ* and length *L*, and obtain the horizontal P_SFH_ and vertical P_SFV_ motion components by decomposing *θ* and *L*.
3. Extend the edge to obtain the extension image of $\left(M+\frac{P_{SFH}}{2}\right)\times \left(N+\frac{P_{SFV}}{2}\right)$.
4. Add the optimal window to the edge extension image.
5. Detect the knife-edge function to get the system degradation function.
6. Use Wiener filtering to get an image free of blurs, and truncate the border to obtain the restored image.

**Fig 4:**
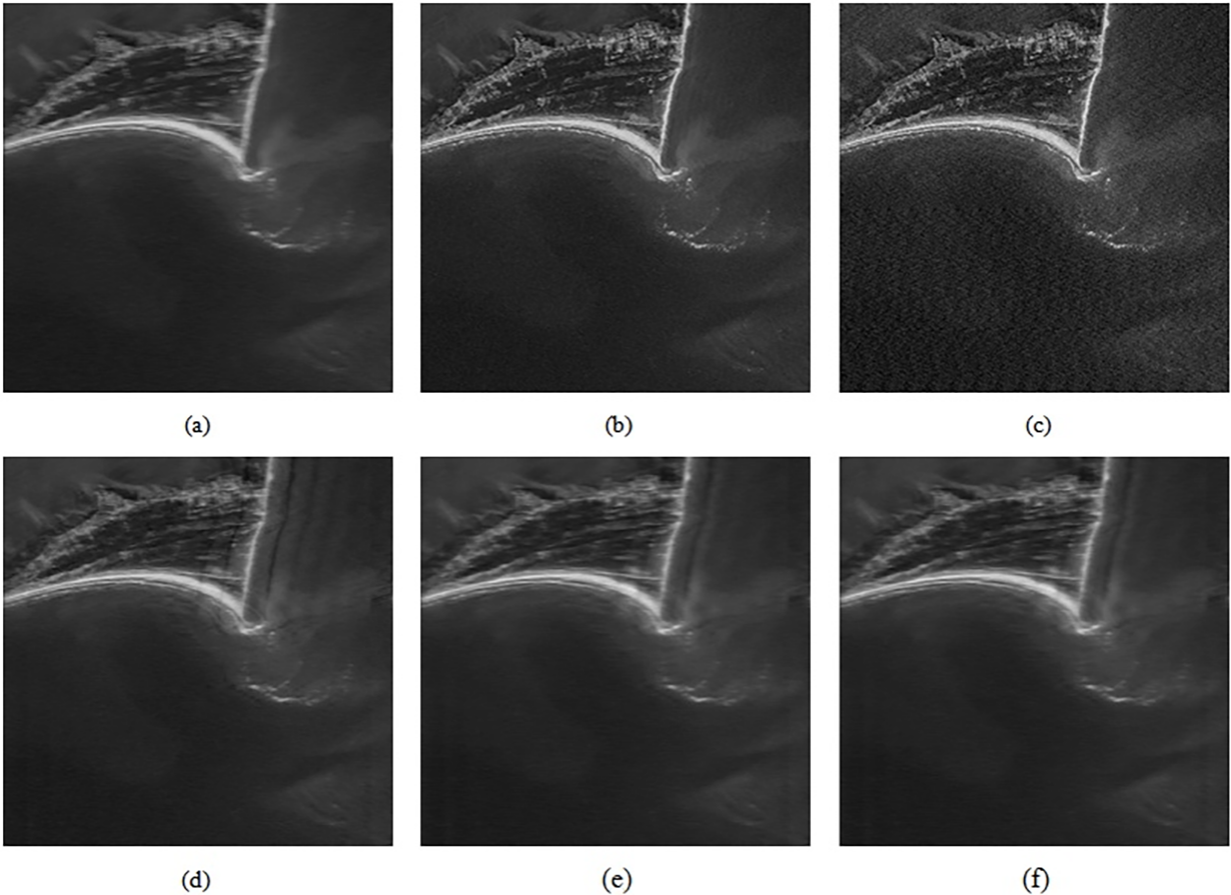
The deblurred performances of different methods on the first test image. (a) The motion-blurred simulation image; (b) the final restoration result based on the proposed technique after edge correction; (c) the restoration result based on rectangular PSF; (d) the restoration result based on traditional Wiener filtering; (e) the restoration result based on traditional Lucy-Richardson; (f) the restoration result based on traditional Blind-deconvolution.

**Fig 5:**
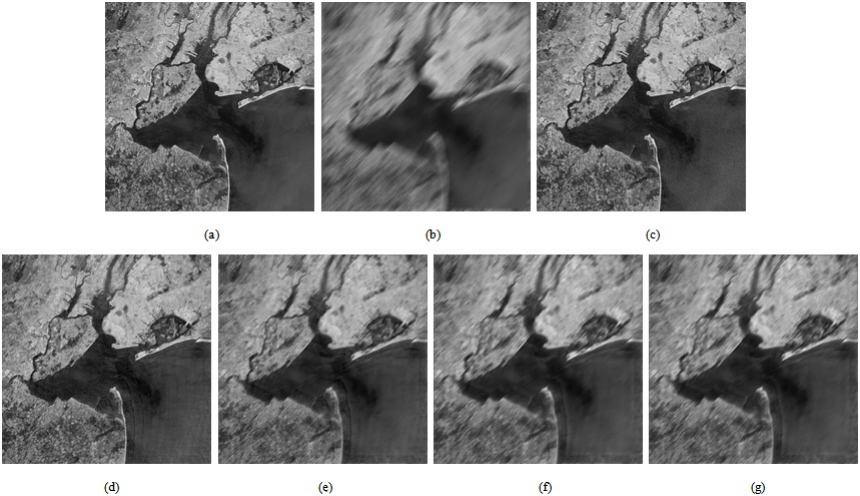
The deblurred performances of different methods on the second test image. (a) The source image; (b) the motion-blurred simulation image; (c) the final restoration result based on the proposed technique; (d) the restoration result based on rectangular PSF; (e) the restoration result based on traditional Wiener filtering; (f) the restoration result based on traditional Lucy-Richardson; (g) the restoration result based on traditional Blind-deconvolution.

We use simulations to evaluate the proposed algorithm on sample datasets from two application scenarios. The images in the first dataset consist of images with horizontal motion-blur (*L* = 30, *θ* = 0°), while the images in the second dataset are original and have motion-blur in an arbitrary direction (*L* = 30, *θ* = 45°). This is shown in Figs 4A, 5A and 5B. Fig 4G–4I) show the process and restored versions of the images in Fig 4A. Fig 5E–5G are the fused images for Fig 5A and 5B, respectively. The images shown in Fig 4G and 4H and Fig 5E–5G are listed below (S2 Fig).

○ The restoration result based on the proposed method (edge correction),
○ The restoration result based on rectangular PSF
○ The restoration result based on traditional Wiener filtering,
○ The restoration result based on traditional Lucy-Richardson, and
○ The restoration result based on traditional Blind-deconvolution.

**Fig 6:**
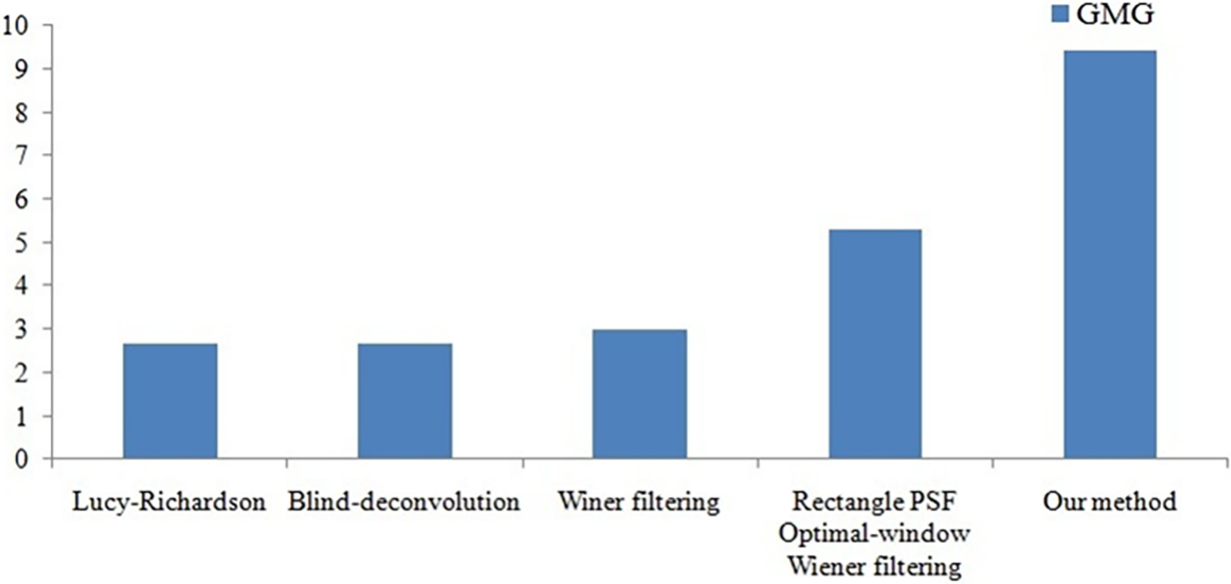
Evaluation of different fusion methods of Fig 4.

**Fig 7:**
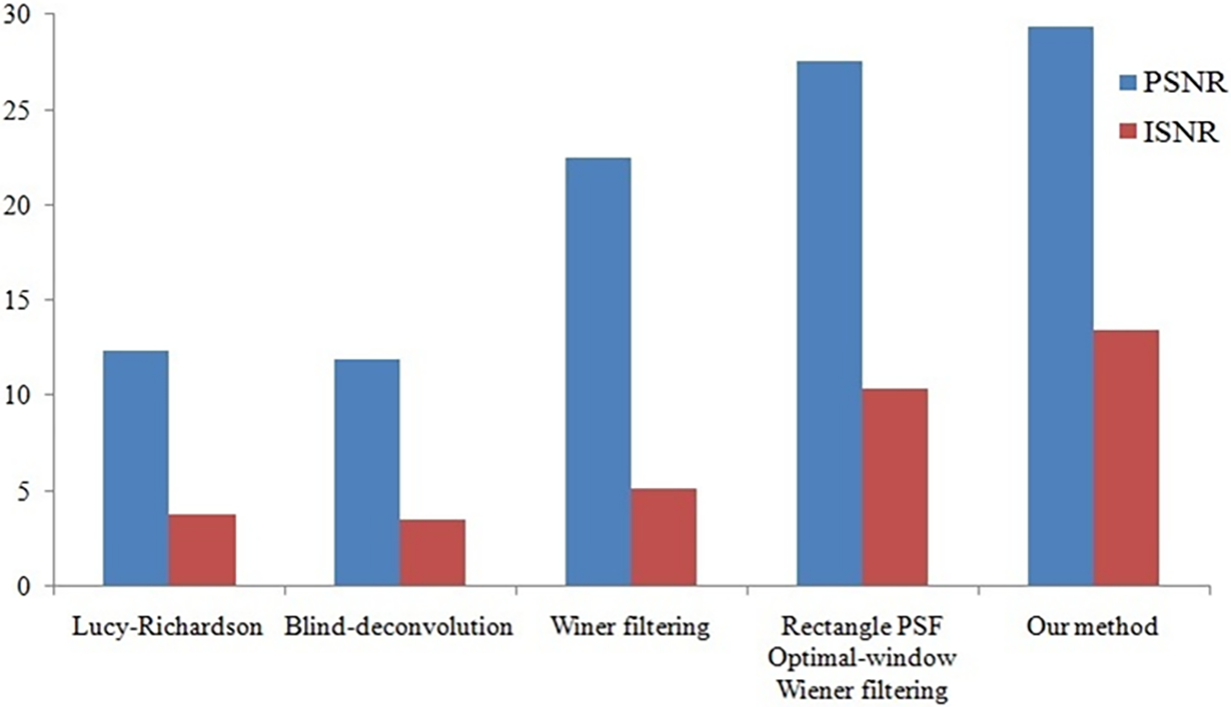
Evaluation of different fusion methods of Fig 5.

Different methods are used to restore Figs 4 and 5 respectively, and the parameters obtained are shown in Figs 6 and 7.

We use three metrics (*GMG*, *PSNR*, and *ISNR*) to evaluate the restoration effect. In the case of the motion-blurred image whose original sharp image is not known (Fig 4), we use *GMG* to evaluate the restoration effect. The larger the *GMG* value, the clearer the image (S1 Table). In the case of the motion-blurred image for which we have clear image (Fig 5), we use *PSNR* and *ISNR to* evaluate the restoration effect. The larger the *PSNR* and *ISNR* values, the greater the improvement (S2 Table).

From Figs 6 and 7, we can see that the performance of the method proposed in this paper is better than that of other techniques. The resolution and contrast parameters are improved, the details are clear, and there is an overall improvement in the image quality.

## Conclusion

This paper proposed an image restoration method using knife-edge function and optimal window Wiener filtering. We constructed a knife-edge function as the PSF to estimate the degradation function without prior knowledge of the specific degradation model. Experiments demonstrated the effectiveness of the proposed technique when compared with other restoration methods.

It is significant to explore the blurred image restoration method to improve the effects of the deblurring without knowledge of the specific degradation model. In the future, we plan to extend the proposed method by integrating more sampling and develop more advanced filtering rules.

## Supporting information

S1 Fig Schematic depiction of the algorithm design used in our method. See Proposed image restoration technique for details of sample description.
(TIF)

S2 Fig Schematic depiction of the experimental design used in the current study. See Proposed image restoration technique for details of sample description.
(TIF)

S1 Table GMG results with five methods of Fig 4. GMG is one of evaluation metrics of restoration effect to the motion-blurred image whose original image is not known, calculated GMG of the restored images from different deblurring.
(DOCX)

S2 Table PSNR and ISNR results with five methods of Fig 5. PSNR and ISNR is two of evaluation metrics of restoration effect to the motion-blurred image whose original sharp image is known, calculated PSNR and ISNR between the original and restored images after different deblurring.
(DOCX)
