# Supplementary figures and images for: Blurred image restoration using knife-edge function and optimal window Wiener filtering
Source: PLoS One. 2018 Jan 29;13(1):e0191833. doi: 10.1371/journal.pone.0191833 (PMC5788387; doi:10.1371/journal.pone.0191833)

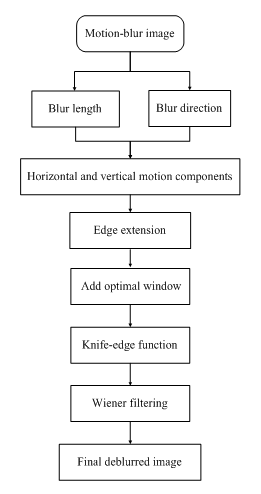

Supplement: S1 Fig — See Proposed image restoration technique for details of sample description. (TIF) [file pone.0191833.s001.tif]

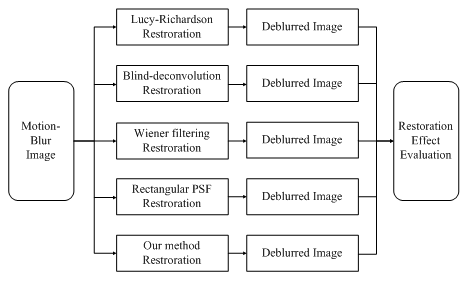

Supplement: S2 Fig — See Proposed image restoration technique for details of sample description. (TIF) [file pone.0191833.s002.tif]
