# Supplementary material for: Blurred image restoration using knife-edge function and optimal window Wiener filtering
Source: PLoS One. 2018 Jan 29;13(1):e0191833. doi: 10.1371/journal.pone.0191833 (PMC5788387; doi:10.1371/journal.pone.0191833)
Supplement: S1 Table — GMG is one of evaluation metrics of restoration effect to the motion-blurred image whose original image is not known, calculated GMG of the restored images from different deblurring. (DOCX) [file pone.0191833.s003.docx]

S1 Table. GMG results with five methods of Fig 4

| **Methods** | **GMG** |
| --- | --- |
| Lucy-Richardson | 2.6514 |
| Blind-deconvolution | 2.6514 |
| Winer filtering | 2.9742 |
| Rectangle PSF Optimal-window Wiener filtering | 5.2904 |
| Our method | 9.4245 |
