# Supplementary material for: Blurred image restoration using knife-edge function and optimal window Wiener filtering
Source: PLoS One. 2018 Jan 29;13(1):e0191833. doi: 10.1371/journal.pone.0191833 (PMC5788387; doi:10.1371/journal.pone.0191833)
Supplement: S2 Table — PSNR and ISNR is two of evaluation metrics of restoration effect to the motion-blurred image whose original sharp image is known, calculated PSNR and ISNR between the original and restored images after different deblurring. (DOCX) [file pone.0191833.s004.docx]

S2 Table. PSNR and ISNR results with five methods of Fig 5

| **Methods** | **PSNR** | **ISNR** |
| --- | --- | --- |
| Lucy-Richardson | 12.3769 | 3.7119 |
| Blind-deconvolution | 11.9026 | 3.4415 |
| Winer filtering | 22.4478 | 5.0904 |
| Rectangle PSF Optimal-window Wiener filtering | 27.5437 | 10.3762 |
| Our method | 29.3981 | 13.4245 |
